# Phylogenetic analysis of canine distemper virus in South African wildlife

**DOI:** 10.1371/journal.pone.0199993

**Published:** 2018-07-18

**Authors:** Angelika K. Loots, Prudent S. Mokgokong, Emily Mitchell, Estelle H. Venter, Antoinette Kotze, Desiré Lee Dalton

**Affiliations:** 1 National Zoological Garden, South African National Biodiversity Institute, Pretoria, South Africa; 2 Department of Veterinary Tropical Diseases, Faculty of Veterinary Science, University of Pretoria, Onderstepoort, South Africa; 3 College of Public Health, Medical and Veterinary Sciences, James Cook University, Townsville, Australia; 4 Genetics Department, University of the Free State, Bloemfontein, Free State, South Africa; 5 Department of Zoology, University of Venda, Thohoyandou, South Africa; Keele University Faculty of Natural Sciences, UNITED KINGDOM

## Abstract

Canine distemper virus (CDV) causes a severe contagious disease in a broad range of hosts. This is the first study to genetically characterise CDV strains from four different wildlife species in South Africa. The phylogenetic diversity of CDV is examined, using the haemagglutinin gene. The South African wildlife CDV isolates showed a high degree of similarity to CDV in South African domestic dogs. Phylogenetic analyses confirmed the presence of 12 geographical lineages with CDV strains from South African wildlife falling within the Southern African lineage. The study reveals two possible co-circulating sub-genotypes corresponding to the northern and southern regions of South Africa respectively. CDV strains from the non-canid species were distinct, but similar to CDV isolates from domestic dog and wild canids. Residues at amino acid sites of the SLAM binding region support the notion that CDV strains encoding 519I / 549H are better adapted to non-canid species than canid species. The amino acids present at site 530 are conserved regardless of host species. Strains from South African wild carnivores showed no difference between host species with all strains presenting 530N. All non-canid strains in this study presented the combination 519I/549H. No evidence of host adaptation or lineage grouping was observed for the Nectin-4 binding region. Further studies should include CDV strains isolated from various hosts from a wider geographical range in South Africa.

## Introduction

Canine distemper virus (CDV; family *Paramyxoviridae*, genus *Morbillivirus*) is a single-stranded, enveloped RNA virus that is reported to cause a severe systemic disease called canine distemper (CD) globally [1]. This contagious disease is characterised by high morbidity and mortality in a taxonomically broad range of immune-naïve hosts, including some non-human primates and several endangered carnivores [2,3]. The development of vaccines against CDV infection in the late 1950s, has considerably reduced the mortality rates, partially controlling the disease in its main reservoir host, namely domestic dogs (*Canis lupus familiaris*) and reducing spill-over of the disease into wildlife species [4–9]. The CDV genome encodes for six structural proteins including the nucleocapsid (N), encapsidating the viral RNA; the phosphor (P) and large protein (L), together forming the transcriptase/replicase complex; the matrix protein (M), important in the budding of virus particles; and the fusion (F) and haemagglutinin (H) protein, important in facilitating viral entry into host cells [10–13]. Based on the genetic variability and the phylogenetic relationship of the H-protein, CDV is classified into several co-circulating genotypes [14]. Genetic lineages largely follow a geographical pattern and include America I, America II, Asia I and II, South America I/ Europe, Europe wildlife, South America II, South America III/Columbian, Arctic-like, Rockborn-like, South Africa and East Africa [11,12,14–21]. These lineages are distinguished on the basis of strains falling within the same clade showing an amino acid divergence of less than 4% in their H-protein region [11,22,23]. Budaszewski *et al*. [24] further suggested that sub-genotypes can be classified within a single clade based on strains with less than 2% divergence within their H-protein. The H-protein is involved in cell tropism and is associated with host shift and adaptability, due to its ability to attach to cellular receptors such as the signalling lymphocyte activation molecule (SLAM, CD150), and Nectin-4 (PVRL4), facilitating viral entry [25,26] (. The importance of an amino acid substitution at site 530 of the CDV H-protein was first highlighted by Seki *et al*. [27]. CDV strains from domestic dog showed a single amino acid substitution at site 530 conferring them the ability to infect both canine or human SLAM-expressing Vero cells as well as B95a (marmoset) cells *in vitro*. Amino acid sites 530 and 549, within the SLAM binding region of the CDV H-protein were later identified to be under positive selection [25]. This was confirmed by Nikolin *et al*. [12], with the addition of site 519 that also showed evidence of episodic positive selection in some genotypes. Differences in residues at these sites have been associated with an adaptation of CDV to non-domestic dog hosts, as is shown with the amino acid substitution of Tyrosine (Y) with Histidine (H) at site 549 [13]. Canine distemper virus strains isolated from Canidae showed a majority of 549Y substitutions, whereas the 549H substitution occurred more often in CDV strains from other carnivore families [25,28]. A combination of amino acids in the CDV H-protein of Isoleucine (I) at site 519 together with H at 549 (519I/549H) was also reported to only occur in infections of non-canid hosts, such as lion (*Panthera leo*) and spotted hyena (*Crocuta crocuta*) [12]. Further investigation of site 530 however found the site to be generally conserved within lineages and that there was no distinguishable association between the amino acid residues at this site and the species infected [12, 29–31],. Conversely, amino acids of the H-protein considered responsible for viral attachment to the Nectin-4 receptor (478, 479, 537, and 539) [32,33] showed no evidence for adaptation to non-canid or canid hosts [12].

Canine distemper virus is thought to have spread from the United Stated to South Africa in the 1920s by way of migration routes [18], leading to the now known South African clade [21]. A recent review by Loots *et al*. [34] focussed on the current research conducted in wildlife, including the latest findings on the causes of host specificity and cellular receptors involved in distemper pathogenesis. It was identified that research into the occurrence and diversity of CDV in wildlife species is severely lacking, specifically in South Africa. Until recently the only research available on other African carnivores infected with CDV originated from Kenya, Tanzania and Botswana [35–38], with the only available CDV H-gene sequences from strains in Tanzania [12]. Thus, in order to obtain a better understanding of CDV dynamics in South Africa, virus strains from wildlife in combination with domestic dogs should be investigated. In this study the phylogenetic diversity of CDV strains recovered in South Africa from four wild carnivore species including lion, African wild dog (*Lycaon pictus*, AWD), spotted- and brown hyena (*Hyaena brunnea*), and one domestic dog is examined (n = 12), using partial amino acid sequence data from the CDV H-protein. Additionally, to examine the molecular adaptation of CDV strains to different carnivore species, residues at amino acid sites of the SLAM and Nectin-4 binding regions on the H-protein were investigated and compared to data available on the National Centre for Biotechnology Information (NCBI) nucleotide database.

## Materials and methods

### Samples

**Fig 1:**
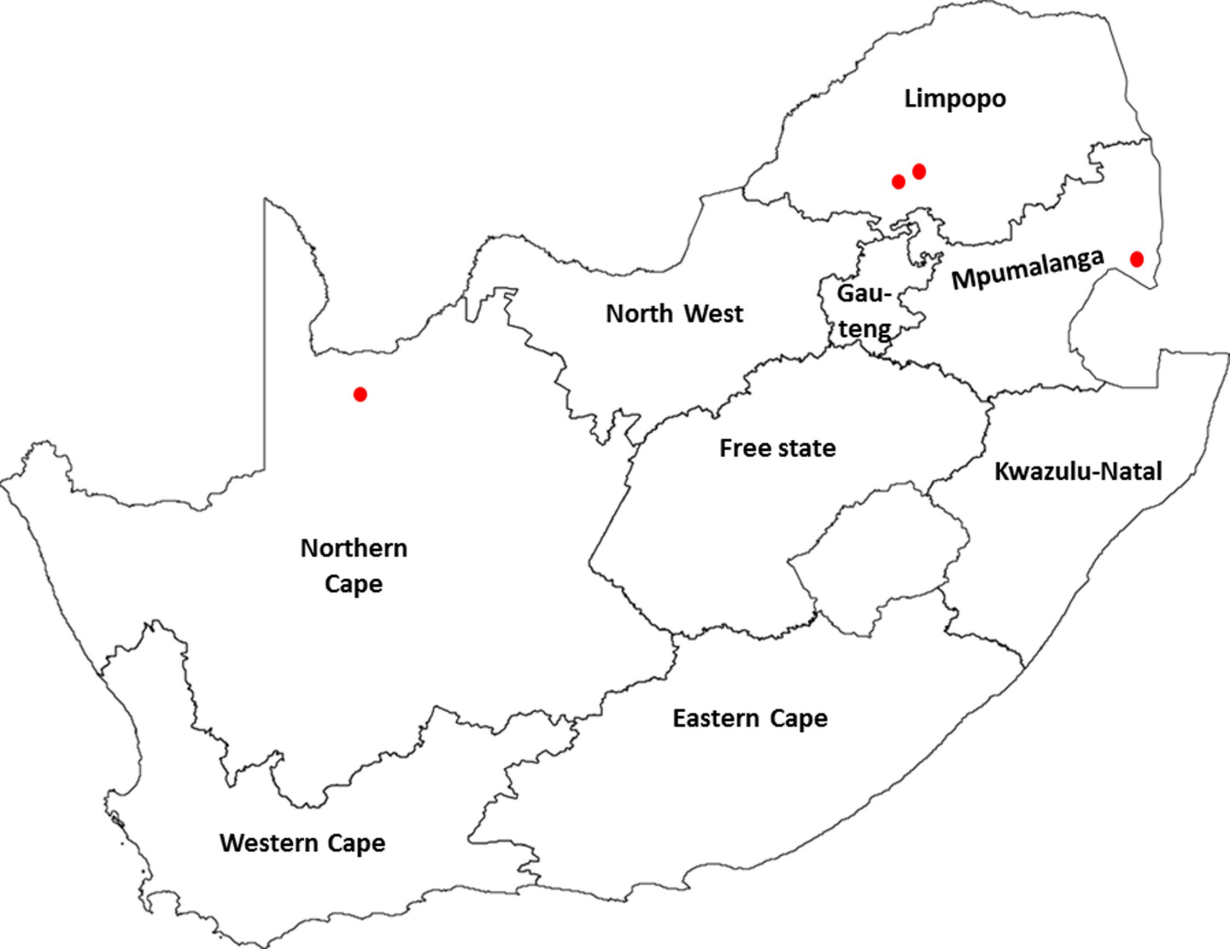
Map of South Africa depicting the different regions were canine distemper virus was isolated from wildlife in 2015/2016.

Canine distemper virus strains were recovered from three different regions in South Africa and were sampled from AWD and domestic dog from the Tswalu Kalahari Reserve, Northern Cape Province; AWD from Kruger National Park (KNP), Mpumalanga Province; brown hyena, lion and spotted hyena from Welgevonden Nature Reserve and a neighbouring nature reserve, Limpopo Province (Table 1, Fig 1). Samples were collected from animals that succumbed due to various clinical signs associated with CDV. Initial positive diagnosis was confirmed by physical examination, typical histopathology and immunohistochemical staining of formalin-fixed paraffin-embedded samples. Ethical approval was obtained from the Animal Ethics Committee, University of Pretoria, South Africa (V072-14) and the National Zoological Gardens of South Africa Research, Ethics and Scientific Committee (P14/26). All samples were obtained under Section 20 permit from the Department of Agriculture, Forestry and Fisheries, South Africa.

**Table 1:** Canine distemper virus strains from wild carnivores and one domestic dog isolated from South Africa in the summer/autumn months of 2015/2016.

Host species	Location	Year sampled	Tissue type	Sequence label	Accession number
African wild dog	Kruger National Park, Mpumalanga, South Africa	2016	Lung	Z1_African wild dog_Kruger	MF467742
African wild dog	Kruger National Park, Mpumalanga, South Africa	2016	Brain	Z2_ African wild dog _Kruger	MF467740
African wild dog	Kruger National Park, Mpumalanga, South Africa	2016	Lung	Z11_ African wild dog _Kruger	MF467743
African wild dog	Kruger National Park, Mpumalanga, South Africa	2016	Lung	Z13_ African wild dog _Kruger	MF467741
African wild dog	Tswalu Kalahari Reserve, Northern Cape, South Africa	2016	Lung	Z9_ African wild dog _Tswalu	MF467739
African wild dog	Tswalu Kalahari Reserve, Northern Cape, South Africa	2016	Lung	Z15_ African wild dog _Tswalu	MF467738
African wild dog	Tswalu Kalahari Reserve, Northern Cape, South Africa	2016	Lung	WT01_ African wild dog _Tswalu	KY971528
Domestic dog	Tswalu Kalahari Reserve, Northern Cape, South Africa	2016	Bladder	Z10_dog_Tswalu	MF467747
Lion	Welgevonden Reserve, Limpopo, South Africa	2015	Kidney	Z6_Lion_Welg	MF467745
Lion	Welgevonden Reserve, Limpopo, South Africa	2015	Spleen	Z7_Lion_Welg	MF467746
Brown Hyena	Welgevonden Reserve, Limpopo, South Africa	2015	Lung	Z4_BHyena_Welg	MF467744
Spotted Hyena	Marakele, Limpopo, South Africa	2016	Lung	WT02_SHyena_Waterberg	KY971532

### RNA extraction

Tissue samples were homogenized in phosphate-buffered saline (PBS) using the Precellys Homogenization system (Bertin Technologies). Subsequent RNA extraction was performed by means of TRIzol LS Reagent (Invitrogen) according to the manufacturer’s instructions and stored at -80°C until used. Two cultured CDV strains commonly used in vaccines, Onderstepoort (OVI) and Nobivac, and RNase-free water were used as positive and negative controls in each reaction cycle, respectively.

### Amplification of the H-gene by nested RT-PCR

Complementary DNA (cDNA) was synthesised with PrimeScript RT Mastermix (Takara) according to the manufacturer’s instructions. Template cDNA was immediately stored at -20°C until used for PCR. Primers were designed based on South African strains previously amplified and sequenced by Woma *et al*. [21]. The H-gene, corresponding to nucleotides 7079–8893 on the CDV genome, was amplified by nested RT-PCR, using a combination of the newly designed primers and primers as previously published, with minor modifications (Table 2). The first round of amplification was achieved using the primer pair RH3-F2 and RH4-R. The inner primer pairs H1F/CDVH1, CDVH2/R1R4, CDVH3/H2RB, CDVH4/CDVH5, CDVH6/CDVH7, H5F/CDVH8, CDVH9/CDVH10, CDVH11/CDVH12, and CDVH13/H7R were used for nested PCR generating overlapping fragments. Amplification conditions consisted of an initial denaturation at 94°C for 3 min followed by 30 cycles of denaturation (94°C for 30 s), annealing (50°C for 30 s) and extension (72°C for 1 min). Final extension was achieved at 72°C for 10 min. All reactions were performed in an ABI 2720 thermal cycler (Applied Biosystems).

**Table 2:** Oligonucleotide primers used in the PCR assays of canine distemper virus H-gene.

Primer	Sequence (5’-3’)	Template length (bp)	Reference
RH3-F2 (RH3-F^a^)	AGG GCT CAG GTA GTC CAG C	Full H-gene	Harder et al. 1996
RH4-R	AAT GCT AGA GAT GGT TTA ATT		Harder et al. 1996
H1F	ATG CTC TCC **Y**AC CAA GAC AA	384	An et al. 2008
CDVH1	GCT CGG ATT GAA GAA GTT TG		Present study
CDVH2	CAA ACT TCT TCA ATC CGA GC	425	Present study
H1R4 (H1R^a^)	CAT RTY ATT CAG CCA CCG TT		An et al. 2008
CDVH3	CAA ACG GTG GCT GAA TGA CA	410	Present study
H2RB	TTT GGT TGC ACA TAG GGT AG		Budaszwenski et al 2014
CDVH4	CGC TCA YCC ATC AGT AGA AA	163	Present study
CDVH5	GTT GCA CAT AGG GTA GGA TT		Present study
CDVH6	AAT CCTA CCC TAT GTG CAA C	159	Present study
CDVH7	CCA TAC CRT CTC CAT TCA GT		Present study
H5F	GGA CAG TTG CCA TCT TAC GG	165	Present study
CDVH8	CTT RGG AGG AAT GGT RAG CC		Present study
CDVH9	ACT GAA TGG AGA YGG TAT GG	159	Present study
CDVH10	CTA GGC GAA AAT GTC AAC AC		Present study
CDVH11	GTG TTG ACA TTT TCG CCT AG	245	Present study
CDVH12	CGT ATA AGA AAT CGT CCG G		Present study
CDVH13	ACG TCG TAG CAA CAT ATG AT	266	Present study
H7R	TCA AGG TYT TGA ACG GTT AC		Present study

Modifications introduced to original published sequence indicated in bold

^a^ Original primer name in reference

### Sequence and phylogenetic analysis of the haemagglutinin gene

Amplicons were visualised by electrophoresis in a 1.5% Tris acetate-EDTA-agarose gel stained with ethidium bromide. Amplified PCR products generated with sets of inner primers were subsequently purified with Exonuclease I and FastAP (Thermo Fisher Scientific Inc.) according to manufacturer’s instructions. Purified products were sequenced on an ABI PRISM 3100 Genetic Analyser using the Big Dye Terminator v.3.1 cycle sequencing kit (Applied Biosystems). Sequencing was conducted in both the forward and reverse direction. Generated overlapping sequences were aligned and contigs constructed in BioEdit Sequence Alignment Editor v.7.2.5 [39]. Resulting contigs were aligned using the multiple alignment method (ClustalW) as implemented in MEGA6 software [40] and visually inspected for nonsense mutations and premature stop codons.

Phylogenetic relationships for the South African CDV H-gene sequences generated in this study, and 193 previously published H-gene sequences from GenBank (http://www.ncbi.nlm.nih.gov) (S1 Table) were inferred by the maximum likelihood (ML) and Bayesian methods. Sequences from GenBank were selected based on previous phylogenetic studies [14]. Potential biased was avoided by selecting sequences from a wide geographic range and time frame. Phocine distemper virus (PDV; Genbank AF479277) was selected as outgroup. The ML trees were constructed using MEGA6 [40]. The general time reversible nucleotide substitution model with gamma distributed rate variation among sites (GTR+G), as selected by MrModeltest v. 2.3 [41], was used. Tree reliability was estimated by 1000 non-parametric bootstrap analyses. A Bayesian phylogram was inferred using the Metropolis-coupled Monte Carlo MarkovChain (MCMC) method as implemented in MrBayes v 3.2.6 [42]. Analyses were initiated from random starting trees using one cold and three incrementally heated chains (0.01) run for 10,000,000 iterations, subsampling every 1000 trees. Twenty per cent of these were discarded as burn-in and the posterior probabilities (PP)calculated from the remaining saved majority rule consensus trees. The GTR+G nucleotide substitution model was also selected. Trees were produced and visualised in FigTree v1.4.0 (http://tree.bio.ed.ac.uk). A subset of aligned H-gene sequences was used to calculate the nucleotide distance matrix and to distinguish CDV lineages based on a 95% similarity at the nucleotide level [22,24].

### Analysis of amino acid sites

Amino acids of the H-protein present at sites 519, 530, and 549 of the SLAM binding region, together with amino acids 478, 479, 493, 537 and 539 of Nectin-4 binding region were determined for the 12 CDV sequences generated in this study, and 177 strains available from GenBank for which information on host, location and date of collection was available (S2 Table). These strains were chosen to represent samples from each genotype as analysed in Ke *et al*. [14].

## Results

### Phylogenetic relationship of the Haemmaglutinin gene

A 1815 base pair (bp) fragment of the CDV Haemmaglutinin gene (H-gene), which includes the SLAM and Nectin-4 binding regions, was amplified and sequenced for 12 clinical specimens obtained from seven AWD, one domestic dog, one spotted hyena, two lions and one brown hyena (Table 1). All sequences were submitted to GenBank under accession numbers MF467738-MF467747. The newly sequenced South African wildlife CDV strains showed a high degree of similarity to CDV in domestic dogs previously isolated from South Africa ranging from 97% to 98% nucleotide identity.

**Fig 2:**
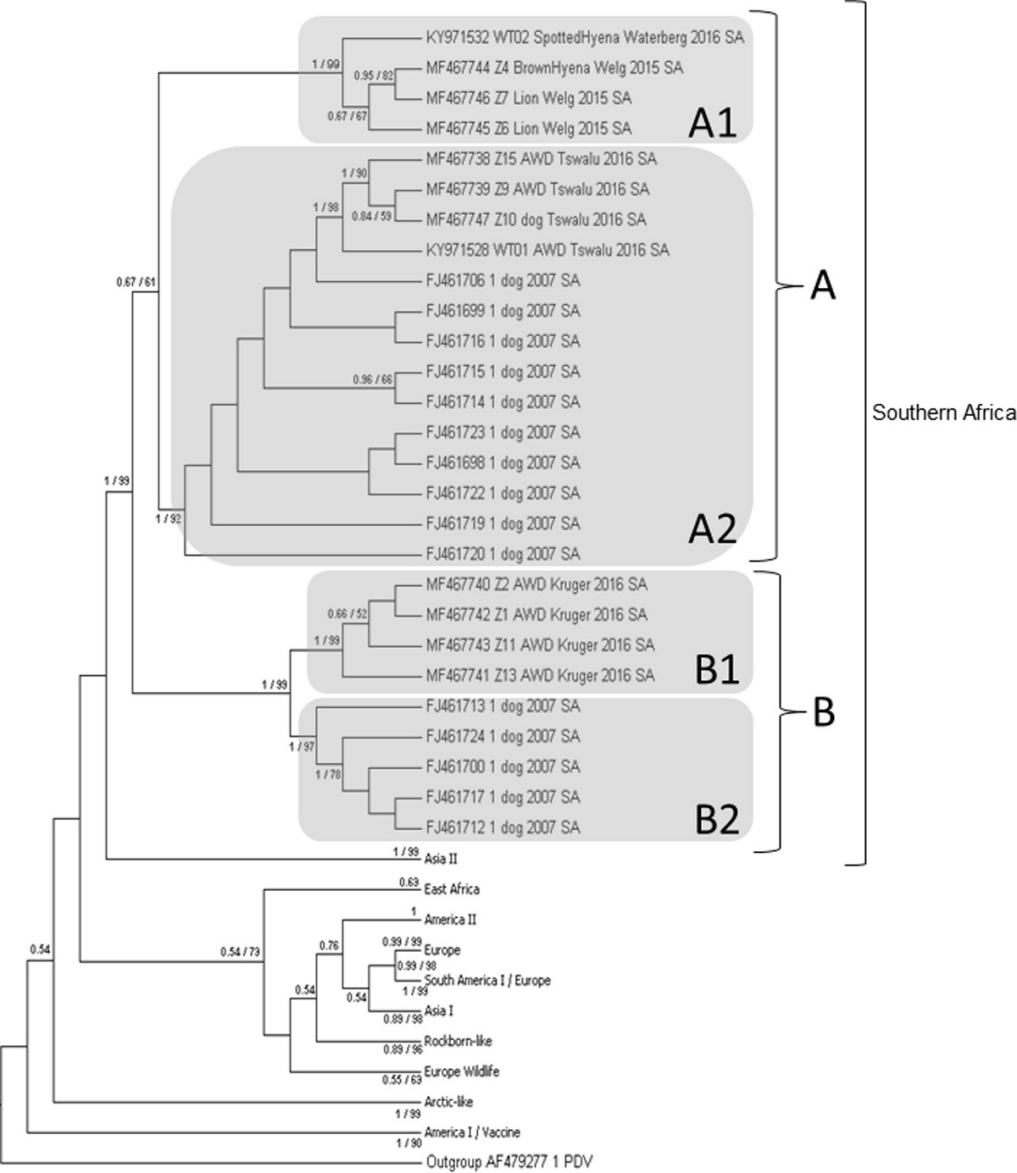
Rooted cladogram of the H-gene sequences of CDV and PDV (outgroup) with nodal support values above 0.5 Bayesian and 50% ML posterior propablities indicated.

Phylogenetic analyses of the H-gene inferred by ML and Bayesian analyses resulted in trees with similar topology. Fig 2 depicts a rooted cladogram of the H-gene sequences of CDV and PDV (outgroup) with nodal support values above 0.5 Bayesian PP and 50% ML bootstrap indicated. Nodal support of 0.9 PP and 70% bootstrap, respectively, are considered as strongly supported. The analyses identified 12 lineages. The outgroup (PDV) first splits into lineage America I (containing most vaccine strains), before splitting into lineage Arctic-like and a group consisting of the lineages Asia I, Rockborn-like, South America II, Europe Wildlife, South America I/Europe, Europe, America II, East Africa, Asia II and Southern Africa. Within the Southern Africa lineage two clades can clearly be defined (indicated as Clade A and Clade B). Clade A splits into two sister clades (A1 and A2). A1 consists of the spotted hyena, brown hyena and lion samples from Limpopo Province and A2 of previously isolated domestic dogs and the newly isolated AWD and dog from the Northern Cape Province. Clade B also splits into two sister clades (B1 and B2). The AWD isolated from Mpumalanga Province group together into B1. B2 exclusively consists of previously isolated domestic dogs. The overall mean genetic distance between unique CDV clusters within the Southern African lineage showed a 3.1% difference between Clade A and Clade B.

### Amino acid variation

Sequenced H-gene fragments from each of the South African field isolates (n = 12) were translated into a 605 amino acid long polypeptide and compared to H-protein strains (n = 177), representing known geographical lineages and various host species (domestic dog, wild canid and non-canid) as sourced from GenBank. The amino acid residue at site 530 was identical (530N) for all South African field isolates obtained in this study, matching all previously sequenced South African domestic dog strains (Table 3). The CDV strain obtained from the domestic dog (MF467747/Z10/dog/2016/SA) in this study specified 519R, 530N and 549Y, identical to former domestic dog CDV strains isolated from South Africa (Table 3). The majority of strains (86%, n = 7) from wild canids in South Africa encoded 519R and most (71%) also presented 549Y. Only one strain from the KNP (MF467742/Z1/African wild dog/2016/SA) encoded with 519I and 549Y (Table 3). Overall analyses of domestic dog and wild canid CDV strains globally showed a majority 519R (99%, n = 102 and 93%, n = 45, respectively) (S2 Table). Of the 102 domestic dog CDV strains analysed 96% presented 549Y and 4% 549H. Wild canids overall (n = 45) had 82% 549Y and 18% 549H. Strains obtained from non-canid species in this study, including two lions, one spotted hyena and one brown hyena, all encoded the amino acid combination of 519I and 549H (n = 4) (Table 3). Overall, only 28% (n = 29) of the strains included in the analysis from non-canid species worldwide (S2 Table) encoded the combination of 519I and 549H. Strains from non-canid species worldwide (S2 Table) encoded residues 519R (62%) or 519I (38%) and 549H (68%) or 549Y (32%).

**Table 3:** Residues at amino acid sites of the SLAM and nectin-4 cell binding regions on the canine distemper virus H-protein isolated in South Africa in 2015/2016. The accession number, host species, year and country of origin are indicated for each strain. Identical amino acids are indicated with a dash (-), varying amino acids are indicated by single letter amino acid codes.

Accession number/species/year/origin	SLAM binding region			Nectin-4 binding region			
	519	530	549	478	479	537	539
**SOUTHERN AFRICA** **Domestic dog**							
MF467747/Z10/dog/2016/SA	R	N	Y	V	L	Y	Y
^a^ FJ461723.1/dog/2007/SA	-	-	-	-	-	-	-
^a^ FJ461698.1/dog/2007/SA	-	-	-	-	-	-	-
^a^ FJ461718.1/dog/2007/SA	-	-	-	-	-	-	-
^a^ FJ461722.1/dog/2007/SA	-	-	-	-	-	-	-
^a^ FJ461704.1/dog/2007/SA	-	-	-	-	-	-	-
^a^ FJ461706.1/dog/2007/SA	-	-	-	-	-	-	-
^a^ FJ461721.1/dog/2007/SA	-	-	-	-	-	-	-
^a^ FJ461695.1/dog/2007/SA	-	-	-	-	-	-	-
^a^ FJ461697.1/dog/2007/SA	-	-	-	-	-	-	-
^a^ FJ461693.1/dog/2007/SA	-	-	-	-	-	-	-
^a^ FJ461703.1/dog/2007/SA	-	-	-	-	-	-	-
^a^ FJ461715.1/dog/2007/SA	-	-	-	-	-	-	-
^a^ FJ461714.1/dog/2007/SA	-	-	-	-	-	-	-
^a^ FJ461699.1/dog/2007/SA	-	-	-	-	-	-	-
^a^ FJ461716.1/dog/2007/SA	-	-	-	-	-	-	-
^a^ FJ461719.1/dog/2007/SA	-	-	-	-	-	-	-
^a^ FJ461720.1/dog/2007/SA	-	-	-	-	-	-	-
^a^ FJ461713.1/dog/2007/SA	-	-	-	-	-	-	-
^a^ FJ461705.1/dog/2007/SA	-	-	-	-	-	-	-
^a^ FJ461696.1/dog/2007/SA	-	-	-	-	-	-	-
^a^ FJ461724.1/dog/2007/SA	-	-	-	-	-	-	-
^a^ FJ461707.1/dog/2007/SA	-	-	-	-	-	-	-
^a^ FJ461711.1/dog/2007/SA	-	-	-	-	-	-	-
^a^ FJ461694.1/dog/2007/SA	-	-	-	-	-	-	-
^a^ FJ461700.1/dog/2007/SA	-	-	-	-	-	-	-
^a^ FJ461717.1/dog/2007/SA	-	-	-	-	-	-	-
^a^ FJ461712.1/dog/2007/SA	-	-	-	-	-	-	-
**Wild canid**							
MF467738/Z15/African wild dog/2016/SA	R	N	Y	V	L	Y	Y
MF467739/Z9/African wild dog /2016/SA	-	-	-	-	-	-	-
MF467740/Z2/African wild dog /2016/SA	-	-	-	-	-	-	-
MF467741/Z13/African wild dog /2016/SA	-	-	-	-	S	-	-
MF467742/Z1/African wild dog /2016/SA	I	-	-	-	-	-	-
MF467743/Z11/African wild dog /2016/SA	-	-	-	-	S	-	-
KY971528/WT01/African wild dog /2016/SA	-	-	-	-	-	-	-
**Non-canid**							
KY971532/WT02/SpottedHyena/2016/SA	I	N	H	V	L	Y	Y
MF467744/Z4/BrownHyena/2016/SA	-	-	-	-	-	-	-
MF467745/Z6/Lion/2015/SA	-	-	-	-	-	-	-
MF467746/Z7/Lion/2015/SA	-	-	-	-	-	-	-

^a^ South African CDV strains isolated by Woma *et al*. (2010) and deposited in GenBank

Amino acid residues thought to be crucial in CDV attachment to the cellular receptor Nectin-4 were generally conserved across species and geographical lineages. All CDV strains isolated in this study presented majority 478V, 479L, 537Y, and 539Y. Two strains from AWD in KNP however resulted in 479S (Table 3). Overall analyses of the Nectin-4 binding sites in CDV strains across geographic lineages also gave majority 478V, 479L, 539Y, and 539Y, although the CDV strain isolated from the javelina (Family: Tyassuidae) from USA in 1995 showed 479W.

## Discussion

The present study characterises CDV from four different wild carnivore species, obtained from three different areas in South Africa. It is also the first report on genetic evidence of CDV in clinical samples from various wildlife species in South Africa. Earlier reports of CDV in South Africa are very limited and it was not until 2010 that CDV strains isolated from domestic dogs were sequenced and phylogenetically characterised [21]. The aforementioned study was however limited to local CDV outbreaks isolated from one species (domestic dog) occurring in one area (Gauteng Province) of South African. The present study reports on the status of CDV infection in South African wildlife and how it relates to currently available genetic sequence data from CDV outbreaks globally.

Phylogenetic analyses of the H-gene sequences of the newly isolated South African strains, together with several globally isolated CDV strains, confirmed the presence of previously described geographical lineages [3,12,14,18] with the newly sequenced CDV strains from South African wildlife falling within the Southern African lineage. This grouping is further supported by the high degree of nucleotide similarity that was observed between the CDV wildlife strains in comparison to the domestic dog strains isolated from South Africa in 2007. Geographical lineages are defined based on a nucleotide difference of 5% between clades [11,24], whereas sub-genotypes can be classified as clades that have a nucleotide difference of more than 2% but less than 5% [24]. Sub-genotypes have thus far only been described in the South America-I / Europe lineage of CDV, showing clear clustering according to distinct geographical areas [24]. The present study revealed the co-circulation of two distinct clades of CDV within the Southern African lineage (Fig 2) with a mean nucleotide difference of 3%, suggesting the co-circulation of two sub-genotypes in South Africa. A correlation between sub-genotype grouping in South Africa and geographical origin of the CDV strains could however not clearly be determined. The first sub-genotype, designated Clade A, comprises sequence data isolated in Limpopo, Northern Cape and Gauteng areas, respectively. The second sub-genotype, designated Clade B, contains mainly isolates from Mpumalanga and Gauteng provinces. It is thus hypothesised that CDV isolates from Clade A are predominantly from the northern parts of South Africa and isolates from Clade B from further south, with both sub-genotypes circulating in Gauteng. This hypothesis should however be confirmed by extending phylogenetic studies to other areas in South Africa.

Focussing on the Southern Africa lineage, it becomes apparent that the phylogenetic relationship of CDV strains isolated from the non-canid species (Felidae and Hyenidae) are distinct, grouping in a separate sister clade (A1), but similar to CDV isolates from both domestic dog and wild canids. Biological and sequence data obtained in previous studies did not indicate the existence of a CDV lineage adapted for non-canine species [12]. All non-canid CDV strains isolated in this study originated from one outbreak in the Limpopo Province area, thus explaining the grouping and supporting previous studies. The addition of a CDV strain isolated from a canid species in the same geographical area will give a better understanding as to the current observed groupings.

Analysis of amino acid substitutions at known functional positions on the SLAM binding region of the CDV H-gene confirmed the importance of sites 519 and 549 in the adaptation of strains to infect various hosts. It also confirms the notion that amino acids present at site 530 in CDV strains infecting various carnivores globally are conserved within lineages regardless of host species. The present analyses showed that the majority of CDV strains exhibit 530G or 530N in the CDV H-protein of wild-, domestic- and non-canine hosts. Strains isolated from South Africa wildlife also showed no difference between host species with all strains presenting 530N, corresponding to the amino acid residue observed in previously isolated domestic dogs from South Africa. Our analyses further confirms the notion of genetic drift at site 530 in certain CDV strains towards A or V in lineages Asia I and Europe wildlife, respectively [25,29].

The arrangement of amino acid residues at site 549 of the CDV H-protein differed in canid and non-canid species, with strains from canids (both domestic and wild) showing a clear bias towards 549Y. CDV strains from non-canid species globally however were equally likely to exhibit H or Y at site 549. These findings are consistent with previous studies and supports the assumption that both canids and non-canid hosts are just as likely to encounter CDV strains with 549Y or 549H, but that canids are more likely to be infected by CDV strains with 549Y [25,28]. This is also consistent with the findings of Nikolin *et al*.[30] that showed an *in vitro* antagonistic pleiotropic effect of site 549, with CDV strains encoding 549Y performing significantly better in cells expressing dog SLAM receptors than those encoding 549H. Nikolin *et al*. [30] also demonstrated a higher performance of CDV-H proteins encoding 549H in celld expressing lion SLAM receptors. The current study presents the first evidence of CDV strains with 549H in the Southern Africa lineage; with all non-canid strains isolated in this study presenting residue H at this site. The current study also reports the presence of the amino acid residue combination 519I/549H on the CDV H-protein isolated from three non-canid species (lion, spotted- and brown hyena). This is consistent with the findings of Nikolin *et al*. [12] that showed strains encoding 519I/549H causing fatal CDV infection only in non-canid hosts during the 1993/1994 Serengeti epidemic.

No evidence of host adaptation or lineage grouping was observed in the four amino acid H-protein sites of the Nectin-4 binding region in CDV. This is consistent with previous reports by Langedijk *et al*. [32]; Sawatsky *et al*. [33]. Sites 478, 537 and 539 were all conserved. However, a CDV strain isolated in a Javelina (Family: Tyassuidae) showed 479W. This could be an indication of site 479 as significant in CDV spread to other mammals outside the order Carnivora, but will have to be substantiated with more data from non-carnivore hosts infected with CDV. As such our data supports Nikolin *et al*. [12] in the notion that residues responsible for the binding of CDV to Nectin-4 have no influence on host adaptation.

In conclusion, the current study presents the first sequence data of CDV infections in South African wild carnivores. The presence of one CDV lineage circulating in South Africa is confirmed, with all wildlife isolates grouping within the Southern African lineage. The study also reveals two possible co-circulating sub-genotypes with a possible geographical pattern at regional level; however more data is needed to confirm this association. The importance of the amino acid residue combination at site 519 and 549 on the SLAM binding region of CDV H-gene in non-canid hosts is also revealed. Conclusions are however limited to available sequence data and in the South African lineage there is a clear bias towards CDV strains isolated in domestic dogs from one particular area. Further studies should thus include CDV strains isolated from various hosts from a wider geographical range in South Africa.

## Supporting information

S1 Table H gene sequence isolates used in determining the phylogenetic relationship of canine distemper virus. The accession number, host species, year and country of origin (when available) are indicated for each strain. South African strains isolated for this study indicated with asterisk (*).
(DOCX)

S2 Table Residues at amino acid sites of the SLAM and nectin-4 cell binding regions on the canine distemper virus H-protein, arranged in geographical lineages and host species (domestic dog, wild canid and non-canid). The accession number, host species, year and country of origin are indicated for each strain. South African strains isolated for this study indicated with asterisk (*). Identical amino acids are indicated with a dash (-), varying amino acids are indicated by single letter amino acid codes.
(DOCX)
