# Supplementary material for: Phylogenetic analysis of canine distemper virus in South African wildlife
Source: PLoS One. 2018 Jul 18;13(7):e0199993. doi: 10.1371/journal.pone.0199993 (PMC6051617; doi:10.1371/journal.pone.0199993)
Supplement: S1 Table — The accession number, host species, year and country of origin (when available) are indicated for each strain. South African strains isolated for this study indicated with asterisk (*). (DOCX) [file pone.0199993.s001.docx]

| **Sample name** | **Lineage** | **Family** | **Species** |
| --- | --- | --- | --- |
| *WT02/SpottedHyena/Waterberg/2016/SA | Southern Africa | Hyenidae | Spotted Hyena |
| *Z15/AWD/Tswalu/SA | Southern Africa | Canidae | African wild dog |
| *Z9/AWD/Tswalu/SA | Southern Africa | Canidae | African wild dog |
| *Z2/AWD/Kruger/SA | Southern Africa | Canidae | African wild dog |
| *Z13/AWD/Kruger/SA | Southern Africa | Canidae | African wild dog |
| *Z1/AWD/Kruger/SA | Southern Africa | Canidae | African wild dog |
| *Z11/AWD/Kruger/SA | Southern Africa | Canidae | African wild dog |
| *Z4/BrownHyena/Welg/SA | Southern Africa | Hyenidae | Brown Hyena |
| *Z6/Lion/Welg/SA | Southern Africa | Felidae | Lion |
| *Z7/Lion/Welg/SA | Southern Africa | Felidae | Lion |
| *Z10/dog/Tswalu/2016/SA | Southern Africa | Canidae | Domestic dog |
| *WT01/AWD/Tswalu/2016/SA | Southern Africa | Canidae | African wild dog |
| FJ461723.1/dog/2007/SA | Southern Africa | Canidae | Domestic dog |
| FJ461722.1/dog/2007/SA | Southern Africa | Canidae | Domestic dog |
| FJ461706.1/dog/2007/SA | Southern Africa | Canidae | Domestic dog |
| FJ461715.1/dog/2007/SA | Southern Africa | Canidae | Domestic dog |
| FJ461699.1/dog/2007/SA | Southern Africa | Canidae | Domestic dog |
| FJ461719.1/dog/2007/SA | Southern Africa | Canidae | Domestic dog |
| FJ461720.1/dog/2007/SA | Southern Africa | Canidae | Domestic dog |
| FJ461713.1/dog/2007/SA | Southern Africa | Canidae | Domestic dog |
| FJ461724.1/dog/2007/SA | Southern Africa | Canidae | Domestic dog |
| FJ461700.1/dog/2007/SA | Southern Africa | Canidae | Domestic dog |
| FJ461717.1/dog/2007/SA | Southern Africa | Canidae | Domestic dog |
| FJ461712.1/dog/2007/SA | Southern Africa | Canidae | Domestic dog |
| JN812975.1/lion/1994/Tanzania | East Africa | Felidae | Lion |
| KC916716.1/bat-earedfox/1994/Tanzania | East Africa | Canidae | Bat-eared fox |
| JN812976.1/dog/1994/Tanzania | East Africa | Canidae | Domestic dog |
| KC916715.1/AWD/2007/Tanzania | East Africa | Canidae | African wild dog |
| KC916714.1/goldenjackal/2011/Tanzania | East Africa | Canidae | Golden Jackal |
| KC916717.1/spottedhyena/1994/Tanzania | East Africa | Hyenidae | Spotted Hyena |
| Z47760.1//Greenlandic/dog/1995/Denmark | Arctic-like | Canidae | Domestic dog |
| Z47763.1/blackleopard/1995/Denmark | America II | Felidae | Black leopard |
| Z47765.1/raccoon/1995/Denmark | America II | Procyonidae | Raccoon |
| Z47764.1/javelina/1995/Denmark | America II | Tayassuidae | Javelina |
| Z47762.1/dog/1995/Denmark | America II | Canidae | Domestic dog |
| HM563057.1/wolf/Portugal/1998 | Europe | Canidae | Wolf |
| HM563058.1/wolf/2008/Portugal | Europe | Canidae | Wolf |
| HM563059.1/dog/2007/Portugal | Europe | Canidae | Domestic dog |
| Z54156.1/Chineseleopard/1995/Netherlands | America II | Felidae | Chinese leopard |
| FJ416339.1/fox/2008/Germany | Europe | Canidae | Fox |
| FJ416338.1/badger/2008/Germany | Europe | Mustelidae | Badger |
| JN153020.1/raccoon/2007/Germany | Europe Wildlife | Procyonidae | Raccoon |
| JN153023.1/raccoon/2007/Germany | Europe Wildlife | Procyonidae | Raccoon |
| JN153025.1/redfox/2008/Germany | Europe | Procyonidae | Raccoon |
| JN153024.1/redfox/2008/Germany | Europe | Procyonidae | Raccoon |
| GQ214373.2/dog/2003/Austria | Arctic-like | Canidae | Domestic dog |
| GQ214374.2/badger/2006/Austria | Europe Wildlife | Mustelidae | Badger |
| GQ214369.2/stonemartin/2007/Austria | Europe Wildlife | Mustelidae | Stone martin |
| GQ214376.2/dog/2002/Austria | Europe | Canidae | Domestic dog |
| GQ214378.2/dog/2002/Austria | Europe | Canidae | Domestic dog |
| DQ226088.1/dog/2005/Italy | Arctic-like | Canidae | Domestic dog |
| DQ226087.1/dog/2005/Italy | Arctic-like | Canidae | Domestic dog |
| DQ228166.1/dog/2005/Italy | Europe Wildlife | Canidae | Domestic dog |
| DQ494317.1/dog/2006/Italy | Europe | Canidae | Domestic dog |
| DQ494319.1/dog/2006/Italy | Europe | Canidae | Domestic dog |
| HM120874.1/redfox/2009/Italy | Europe | Canidae | Red fox |
| DQ494318.1/dog/2006/Italy | Europe | Canidae | Domestic dog |
| GU001863.1/Iberianlynx/2005/Spain | Europe | Felidae | Iberian lynx |
| GU001864.1/Iberianlynx/2005/Spain | Europe | Felidae | Iberian lynx |
| DQ889177.1/dog/2006/Hungary | Europe | Canidae | Domestic dog |
| AY542312.2/racoon/2004/USA | America I | Procyonidae | Raccoon |
| AY526496.1/raccoon/2004/USA | America II | Procyonidae | Raccoon |
| AY438597.1/raccoon/2003/USA | America II | Procyonidae | Raccoon |
| AY649446.1/raccoon/2004/USA | America II | Procyonidae | Raccoon |
| AY548111.1/racoon/2004/USA | America I | Procyonidae | Raccoon |
| AY964112.1/dog/2005/USA | Arctic-like | Canidae | Domestic dog |
| FJ392652.1/dog/2003/Argentina | South America I / Europe | Canidae | Domestic dog |
| FJ392651.1/dog/2005/Argentina | South America II | Canidae | Domestic dog |
| KC257464.1/dog/2010/Argentina | South America II | Canidae | Domestic dog |
| FJ011005.1/dog/2005/Argentina | South America II | Canidae | Domestic dog |
| JN215476.1/dog/2009/Uruguay | South America I / Europe | Canidae | Domestic dog |
| JN215475.1/dog/2008/Uruguay | South America I / Europe | Canidae | Domestic dog |
| JN215473.1/dog/2007/Uruguay | South America I / Europe | Canidae | Domestic dog |
| JN215477.1/dog/2009/Uruguay | South America I / Europe | Canidae | Domestic dog |
| JN215474.1/dog/2008/Uruguay | South America I / Europe | Canidae | Domestic dog |
| EU098105.1/dog/2007/Brazil | South America I / Europe | Canidae | Domestic dog |
| EU098103.1/dog/2007/Brazil | South America I / Europe | Canidae | Domestic dog |
| EU098104.1/dog/2007/Brazil | South America I / Europe | Canidae | Domestic dog |
| EU098102.1/dog/2007/Brazil | South America I / Europe | Canidae | Domestic dog |
| HQ403645.1/dog/2009/China | America I | Canidae | Domestic dog |
| EF445052.1/fox/2007/China | Arctic-like | Canidae | Fox |
| GQ332531.1/dog/2008/China | America I | Canidae | Domestic dog |
| JN381191.1/dog/2011/China | Asia I | Canidae | Domestic dog |
| EF445053.1/fox/2007/China | Asia I | Canidae | Fox |
| FJ405223.1/monkey/2008/China | Asia I | Primates | Monkey |
| FJ405224.1/monkey/2008/China | Asia I | Primates | Monkey |
| EU325721.1/fox/2007/China | Asia I | Canidae | Fox |
| HM448829.1/fox/2009/China | Asia I | Canidae | Fox |
| HM448831.1/fox/2009/China | Asia I | Canidae | Fox |
| HM448832.1/raccoondog/2009/China | Asia I | Canidae | Raccoon dog |
| FJ810213.1/fox/2008/China | Asia I | Canidae | Fox |
| EU325728.1/raccoondog/2007/China | Asia I | Canidae | Raccoon dog |
| EU564813.1/dog/2007/China | Asia I | Canidae | Domestic dog |
| EU564812.1/dog/2007/China | Asia I | Canidae | Domestic dog |
| EU684265.1/dog/2007/China | Asia I | Canidae | Domestic dog |
| DQ922630.1/fox/2006/China | Asia I | Canidae | Domestic dog |
| GQ332530.1/dog/2008/China | Asia I | Canidae | Domestic dog |
| FJ851452.1/dog/2008/China | Asia I | Canidae | Domestic dog |
| FJ848530.1/dog/2008/China | Asia I | Canidae | Domestic dog |
| FJ851456.1/dog/2008/China | Asia I | Canidae | Domestic dog |
| FJ535063.1/dog/2008/China | Asia I | Canidae | Domestic dog |
| HM623891.1/dog/2009/China | Asia I | Canidae | Domestic dog |
| HM623893.1/dog/2009/China | Asia I | Canidae | Domestic dog |
| EU325724.1/mink/2007/China | Asia I | Mustelidae | Mink |
| FJ851454.1/dog/2008/China | Asia I | Canidae | Domestic dog |
| EU325723.1/mink/2007/China | Asia I | Mustelidae | Mink |
| EU379560.1/mink/2007/China | Asia I | Mustelidae | Mink |
| EU325720.1/fox/2007/China | Asia I | Canidae | Fox |
| HM448834.1/fox/2009/China | Asia I | Canidae | Fox |
| EU934233.1/raccoondog/2006/China | Asia I | Canidae | Raccoon dog |
| EU325722.1/fox/2006/China | Asia I | Canidae | Domestic dog |
| EU325726.1/raccoondog/2006/China | Asia I | Canidae | Raccoon dog |
| FJ851450.1/dog/2008/China | Asia I | Canidae | Domestic dog |
| EF445051.1/fox/2007/China | Asia I | Canidae | Fox |
| EF042818.1/raccoondog/2006/China | Asia I | Canidae | Raccoon dog |
| EU325729.1/raccoondog/2007/China | Asia I | Canidae | Raccoon dog |
| FJ848536.1/dog/2008/China | Asia I | Canidae | Domestic dog |
| GQ332534.1/dog/2008/China | Asia I | Canidae | Domestic dog |
| HQ850147.1/dog/2008/China | Asia I | Canidae | Domestic dog |
| HM623895.1/dog/2009/China | Asia I | Canidae | Domestic dog |
| HM448833.1/raccoondog/2009/China | Asia I | Canidae | Raccoon dog |
| HM448830.1/raccoondog/2009/China | Asia I | Canidae | Raccoon dog |
| JF343962.1/dog/2009/China | Asia I | Canidae | Domestic dog |
| HM749644.1/dog/2009/China | Asia I | Canidae | Domestic dog |
| FJ851455.1/dog/2008/China | Asia I | Canidae | Domestic dog |
| EU325731.1/mink/2007/China | Asia I | Mustelidae | Mink |
| EU325730.1/raccoondog/2007/China | Asia I | Canidae | Raccoon dog |
| FJ810215.1/fox/2008/China | Asia I | Canidae | Fox |
| EF445054.1/raccoondog/2007/China | Asia I | Canidae | Raccoon dog |
| EU325727.1/raccoondog/2007/China | Asia I | Canidae | Raccoon dog |
| EU325725.1/mink/2006/China | Asia I | Mustelidae | Mink |
| HQ128601.1/raccoondog/2010/China | Asia I | Canidae | Raccoon dog |
| HQ128600.1/dog/2010/China | Asia I | Canidae | Domestic dog |
| HQ128599.1/dog/2010/China | Asia I | Canidae | Domestic dog |
| FJ848531.1/dog/2008/China | Asia I | Canidae | Domestic dog |
| FJ848533.1/dog/2008/China | Asia I | Canidae | Domestic dog |
| HQ657209.1dog/2010/China | Asia I | Canidae | Domestic dog |
| FJ848532.1/dog/2008/China | Asia I | Canidae | Domestic dog |
| GQ332532.1/dog/2008/China | Asia I | Canidae | Domestic dog |
| FJ810214.1/raccoondog/2008/China | Asia I | Canidae | Raccoon dog |
| EU716075.1/dog/2007/SouthKorea | Asia II | Canidae | Domestic dog |
| EU716074.1/marten/1998/SouthKorea | Asia II | Mustelidae | Marten |
| EU716073.1/dog/1997/SouthKorea | Asia II | Canidae | Domestic dog |
| EU716072.1/dog/2007/SouthKorea | Asia I | Canidae | Domestic dog |
| AB025270.1/dog/1999/Japan | Asia II | Canidae | Domestic dog |
| AB040767/dog/2000/Japan | Asia II | Canidae | Domestic dog |
| AB605890.1/raccoondog/2008/Japan | Asia I | Canidae | Raccoon dog |
| AB619774.1/tiger/2010/Japan | Asia I | Felidae | Tiger |
| AB619775.1/raccoondog/2009/Japan | Asia I | Canidae | Raccoon dog |
| AB605891.1/raccoondog/2007/Japan | Asia I | Canidae | Raccoon dog |
| AB025271.2/dog/1999/Japan | Asia I | Canidae | Domestic dog |
| FJ851453.1/dog/2008/China | Asia I | Canidae | Domestic dog |
| FJ851451.1/dog/2008/China | Asia I | Canidae | Domestic dog |
| EU296492.1/dog/2005/Taiwan | Asia I | Canidae | Domestic dog |
| EU296491.1/dog/2006/Taiwan | Asia I | Canidae | Domestic dog |
| EU296490.1/dog/2005/Taiwan | Asia I | Canidae | Domestic dog |
| EU296485.1/dog/2006/Taiwan | Asia I | Canidae | Domestic dog |
| FJ705234.1/dog/2008/Taiwan | Asia I | Canidae | Domestic dog |
| EU296486.1dog/2006/Taiwan | Asia I | Canidae | Domestic dog |
| DQ191175.1/dog/2004/Taiwan | Asia I | Canidae | Domestic dog |
| EU296481.1/dog/2005/Taiwan | Asia I | Canidae | Domestic dog |
| EU296484.1/dog/2006/Taiwan | Asia I | Canidae | Domestic dog |
| EU296483.1/dog/2005/Taiwan | Asia I | Canidae | Domestic dog |
| FJ705232.1/dog/2008/Taiwan | Asia I | Canidae | Domestic dog |
| EU296488.1/dog/2005/Taiwan | Asia I | Canidae | Domestic dog |
| EU296489.1/dog/2005/Taiwan | Asia I | Canidae | Domestic dog |
| EU296494.1/dog/2007/Taiwan | Asia I | Canidae | Domestic dog |
| EU296487.1/dog/2005/Taiwan | Asia I | Canidae | Domestic dog |
| FJ705233.1/dog/2008/Taiwan | Asia I | Canidae | Domestic dog |
| FJ461710.1/CDV/Canigen | America I |  |  |
| FJ461709.1/CDV/NobivacPuppyDP | America I |  |  |
| FJ461708.1/CDV/GalaxyDA2PPV | America I |  |  |
| FJ461702.1/CDV/VanguardPlus | America I |  |  |
| FJ461701.1/CDV/NobivacDHPPI | America I |  |  |
| AF259552.1/CDV/SnyderHill | America I |  |  |
| EU143737.1/CDV/Onderstepoort | America I |  |  |
| Z35493.1/CDV/Convac | America I |  |  |
| DQ903854.1/CDV/Lederle | America I |  |  |
