# Supplementary material for: Phylogenetic analysis of canine distemper virus in South African wildlife
Source: PLoS One. 2018 Jul 18;13(7):e0199993. doi: 10.1371/journal.pone.0199993 (PMC6051617; doi:10.1371/journal.pone.0199993)
Supplement: S2 Table — The accession number, host species, year and country of origin are indicated for each strain. South African strains isolated for this study indicated with asterisk (*). Identical amino acids are indicated with a dash (-), varying amino acids are indicated by single letter amino acid codes. (DOCX) [file pone.0199993.s002.docx]

|  | **Accession number/species/year/origin** | **SLAM binding region** | | | | **Nectin-4 binding region** | | | | | | |
| --- | --- | --- | --- | --- | --- | --- | --- | --- | --- | --- | --- | --- |
|  |  | **519** | **530** | **549** | **478** | | | **479** | | **537** | **539** | |
|  | **SOUTHERN AFRICA** |  |  |  |  |  |  |  |  |  |  |  |
|  | **Domestic dog** |  |  |  |  |  |  |  |  |  |  |  |
| 1 | *****MF467747/Z10/dog/2016/SA | R | N | Y | V | | L | | Y | | | Y |
| 2 | FJ461723.1/dog/2007/SA | - | - | - | - | | - | | - | | | - |
| 3 | FJ461722.1/dog/2007/SA | - | - | - | - | | - | | - | | | - |
| 4 | FJ461706.1/dog/2007/SA | - | - | - | - | | - | | - | | | - |
| 5 | FJ461715.1/dog/2007/SA | - | - | - | - | | - | | - | | | - |
| 6 | FJ461699.1/dog/2007/SA | - | - | - | - | | - | | - | | | - |
| 7 | FJ461719.1/dog/2007/SA | - | - | - | - | | - | | - | | | - |
| 8 | FJ461720.1/dog/2007/SA | - | - | - | - | | - | | - | | | - |
| 9 | FJ461713.1/dog/2007/SA | - | - | - | - | | - | | - | | | - |
| 10 | FJ461724.1/dog/2007/SA | - | - | - | - | | - | | - | | | - |
| 11 | FJ461700.1/dog/2007/SA | - | - | - | - | | - | | - | | | - |
| 12 | FJ461717.1/dog/2007/SA | - | - | - | - | | - | | - | | | - |
| 13 | FJ461712.1/dog/2007/SA | - | - | - | - | | - | | - | | | - |
|  | **Wild canid** |  |  |  |  | |  | |  | | |  |
| 14 | *****MF467738/Z15/African wild dog/2016/SA | R | N | Y | V | | L | | Y | | | Y |
| 15 | *MF467739/Z9/African wild dog /2016/SA | - | - | - | - | | - | | - | | | - |
| 16 | *MF467740/Z2/African wild dog /2016/SA | - | - | - | - | | - | | - | | | - |
| 17 | *MF467741/Z13/African wild dog /2016/SA | - | - | - | - | | S | | - | | | - |
| 18 | *MF467742/Z1/African wild dog /2016/SA | I | - | - | - | | - | | - | | | - |
| 19 | *MF467743/Z11/African wild dog /2016/SA | - | - | - | - | | S | | - | | | - |
| 20 | *KY971528/WT01/African wild dog /2016/SA | - | - | - | - | | - | | - | | | - |
|  | **Non-canid** |  |  |  |  | |  | |  | | |  |
| 21 | *KY971532/WT02/SpottedHyena/2016/SA | I | N | H | V | | L | | Y | | | Y |
| 22 | *MF467744/Z4/BrownHyena/2016/SA | - | - | - | - | | - | | - | | | - |
| 23 | *MF467745/Z6/Lion/2015/SA | - | - | - | - | | - | | - | | | - |
| 24 | *MF467746/Z7/Lion/2015/SA | - | - | - | - | | - | | - | | | - |
| 25 | **EAST AFRICA** |  |  |  |  | |  | |  | | |  |
|  | **Domestic dog** |  |  |  |  | |  | |  | | |  |
| 26 | JN812976.1/dog/1994/Tanzania | R | D | Y | V | | L | | Y | | | Y |
|  | **Wild canid** |  |  |  |  | |  | |  | | |  |
| 27 | KC916716.1/bat-earedfox/1994/Tanzania | R | D | H | V | | L | | Y | | | Y |
| 28 | KC916715.1/African wild dog/2007/Tanzania | - | - | - | - | | - | | - | | | - |
| 29 | KC916714.1/goldenjackal/2011/Tanzania | - | - | Y | - | | - | | - | | | - |
|  | **Non-canid** |  |  |  |  | |  | |  | | |  |
| 30 | JN812975.1/lion/1994/Tanzania | I | D | H | V | | L | | Y | | | Y |
| 31 | KC916717.1/spottedhyena/1994/Tanzania | - | - | - | - | | - | | - | | | - |
|  | **AMERICA I** |  |  |  |  | |  | |  | | |  |
| 32 | **Domestic dog** |  |  |  |  | |  | |  | | |  |
| 33 | HQ403645.1/dog/2009/China | R | N | H | V | | L | | Y | | | Y |
| 34 | GQ332531.1/dog/2008/China | - | - | - | - | | - | | - | | | - |
|  | **Non-canid** |  |  |  |  | |  | |  | | |  |
| 35 | AY542312.2/racoon/2004/USA | R | N | Y | V | | L | | Y | | | Y |
| 36 | AY548111.1/racoon/2004/USA | - | - | - | - | | - | | - | | | - |
|  | **Vaccine** |  |  |  |  | |  | |  | | |  |
| 37 | FJ461710.1/CDV/Canigen | R | S | H | V | | L | | Y | | | Y |
| 38 | FJ461709.1/CDV/NobivacPuppyDP | - | - | - | - | | - | | - | | | - |
| 39 | FJ461708.1/CDV/GalaxyDA2PPV | - | - | - | - | | - | | - | | | - |
| 40 | FJ461702.1/CDV/VanguardPlus | - | D | Y | - | | - | | - | | | - |
| 41 | FJ461701.1/CDV/NobivacDHPPI | - | - | - | - | | - | | - | | | - |
| 42 | AF259552.1/CDV/SnyderHill | - | N | Y | - | | - | | - | | | - |
| 43 | EU143737.1/CDV/Onderstepoort | - | - | - | - | | - | | - | | | - |
| 44 | Z35493.1/CDV/Convac | - | - | - | - | | - | | - | | | - |
| 45 | DQ903854.1/CDV/Lederle | - | - | - | - | | - | | - | | | - |
|  | **AMERICA II** |  |  |  |  | |  | |  | | |  |
|  | **Domestic dog** |  |  |  |  | |  | |  | | |  |
| 46 | Z47762.1/dog/1995/Denmark | R | G | H | V | | L | | Y | | | Y |
|  | **Non-canid** |  |  |  |  | |  | |  | | |  |
| 47 | Z47763.1/blackleopard/1995/Denmark | R | G | H | V | | L | | Y | | | Y |
| 48 | Z47765.1/raccoon/1995/Denmark | - | - | - | - | | - | | - | | | - |
| 49 | Z54156.1/Chineseleopard/1995/Netherlands | I | - | - | - | | - | | - | | | - |
| 50 | AY526496.1/raccoon/2004/USA | - | - | - | - | | - | | - | | | - |
| 51 | AY438597.1/raccoon/2003/USA | I | - | - | - | | - | | - | | | - |
| 52 | AY649446.1/raccoon/2004/USA | - | R | - | - | | - | | - | | | - |
|  | **ARCTIC-LIKE** |  |  |  |  | |  | |  | | |  |
|  | **Domestic dog** |  |  |  |  | |  | |  | | |  |
| 53 | Z47760.1//Greenlandic/dog/1995/Denmark | R | N | Y | V | | L | | Y | | | Y |
| 54 | GQ214373.2/dog/2003/Austria | - | - | - | - | | - | | - | | | - |
| 55 | DQ226088.1/dog/2005/Italy | - | - | - | - | | - | | - | | | - |
| 56 | DQ226087.1/dog/2005/Italy | - | - | - | - | | - | | - | | | - |
| 57 | AY964112.1/dog/2005/USA | - | - | - | - | | - | | - | | | - |
|  | **Wild canid** |  |  |  |  | |  | |  | | |  |
| 58 | EF445052.1/fox/2007/China | R | N | Y | V | | L | | Y | | | Y |
|  | **ASIA I** |  |  |  |  | |  | |  | | |  |
|  | **Domestic dog** |  |  |  |  | |  | |  | | |  |
| 59 | JN381191.1/dog/2011/China | R | G | Y | V | | L | | Y | | | Y |
| 60 | EU564813.1/dog/2007/China | - | - | - | - | | - | | D | | | - |
| 61 | EU564812.1/dog/2007/China | - | - | - | - | | - | | - | | | - |
| 62 | EU684265.1/dog/2007/China | - | - | - | - | | - | | - | | | - |
| 63 | GQ332530.1/dog/2008/China | - | - | - | - | | - | | - | | | - |
| 64 | FJ851452.1/dog/2008/China | - | - | - | - | | - | | - | | | - |
| 65 | FJ848530.1/dog/2008/China | - | - | - | - | | - | | - | | | - |
| 66 | FJ851456.1/dog/2008/China | - | - | - | - | | - | | - | | | - |
| 67 | FJ535063.1/dog/2008/China | - | - | - | - | | - | | - | | | - |
| 68 | HM623891.1/dog/2009/China | - | A | - | - | | - | | - | | | - |
| 69 | HM623893.1/dog/2009/China | - | A | - | - | | - | | - | | | - |
| 70 | FJ851454.1/dog/2008/China | - | - | - | - | | - | | - | | | - |
| 71 | FJ851450.1/dog/2008/China | - | - | - | - | | - | | - | | | - |
| 72 | FJ848536.1/dog/2008/China | - | - | - | - | | - | | - | | | - |
| 73 | GQ332534.1/dog/2008/China | - | - | - | - | | - | | - | | | - |
| 74 | HQ850147.1/dog/2008/China | - | - | - | - | | - | | - | | | - |
| 75 | HM623895.1/dog/2009/China | - | - | - | - | | - | | - | | | - |
| 76 | JF343962.1/dog/2009/China | - | - | - | - | | - | | - | | | - |
| 77 | HM749644.1/dog/2009/China | - | - | - | - | | - | | - | | | - |
| 78 | FJ851455.1/dog/2008/China | - | - | - | - | | - | | - | | | - |
| 79 | HQ128600.1/dog/2010/China | - | - | - | - | | - | | - | | | - |
| 80 | HQ128599.1/dog/2010/China | - | - | - | - | | - | | - | | | - |
| 81 | FJ848531.1/dog/2008/China | - | - | - | - | | - | | - | | | - |
| 82 | FJ848533.1/dog/2008/China | - | - | - | - | | - | | - | | | - |
| 83 | HQ657209.1dog/2010/China | - | - | - | - | | - | | - | | | - |
| 84 | FJ848532.1/dog/2008/China | - | - | - | - | | - | | - | | | - |
| 85 | GQ332532.1/dog/2008/China | - | - | - | - | | - | | - | | | - |
| 86 | EU716072.1/dog/2007/SouthKorea | - | - | - | - | | - | | - | | | - |
| 87 | AB025271.2/dog/1999/Japan | - | - | - | - | | - | | - | | | - |
| 88 | FJ851453.1/dog/2008/China | - | A | - | - | | - | | - | | | - |
| 89 | FJ851451.1/dog/2008/China | - | A | - | - | | - | | - | | | - |
| 90 | EU296492.1/dog/2005/Taiwan | - | - | - | - | | - | | - | | | - |
| 91 | EU296491.1/dog/2006/Taiwan | - | - | - | - | | - | | - | | | - |
| 92 | EU296490.1/dog/2005/Taiwan | - | - | - | - | | - | | - | | | - |
| 93 | EU296485.1/dog/2006/Taiwan | - | - | - | - | | - | | - | | | - |
| 94 | FJ705234.1/dog/2008/Taiwan | - | - | - | - | | - | | - | | | - |
| 95 | EU296486.1/dog/2006/Taiwan | - | - | - | - | | - | | - | | | - |
| 96 | DQ191175.1/dog/2004/Taiwan | - | - | - | - | | - | | - | | | - |
| 97 | EU296481.1/dog/2005/Taiwan | - | - | H | - | | - | | - | | | - |
| 98 | EU296484.1/dog/2006/Taiwan | - | - | - | - | | - | | - | | | - |
| 99 | EU296483.1/dog/2005/Taiwan | - | - | - | - | | - | | - | | | - |
| 100 | FJ705232.1/dog/2008/Taiwan | - | - | - | - | | - | | - | | | - |
| 101 | EU296488.1/dog/2005/Taiwan | - | - | - | - | | - | | - | | | - |
| 102 | EU296489.1/dog/2005/Taiwan | - | - | - | - | | - | | - | | | - |
| 103 | EU296494.1/dog/2007/Taiwan | - | - | - | - | | - | | - | | | - |
| 104 | EU296487.1/dog/2005/Taiwan | - | - | - | - | | - | | - | | | - |
| 105 | FJ705233.1/dog/2008/Taiwan | - | - | - | - | | - | | - | | | - |
|  | **Wild canid** |  |  |  |  | |  | |  | | |  |
| 106 | EU325722.1/fox/2006/China | R | G | Y | V | | L | | Y | | | Y |
| 107 | DQ922630.1/fox/2006/China | - | - | - | - | | - | | - | | | - |
| 108 | EF445053.1/fox/2007/China | - | - | - | - | | - | | - | | | - |
| 109 | EU325721.1/fox/2007/China | - | - | - | - | | - | | - | | | - |
| 110 | HM448829.1/fox/2009/China | - | - | H | - | | - | | - | | | - |
| 111 | HM448831.1/fox/2009/China | - | - | - | - | | - | | - | | | - |
| 112 | HM448832.1/raccoondog/2009/China | - | - | - | - | | - | | - | | | - |
| 113 | FJ810213.1/fox/2008/China | - | - | - | - | | - | | - | | | - |
| 114 | EU325728.1/raccoondog/2007/China | - | - | - | - | | - | | - | | | - |
| 115 | EU325720.1/fox/2007/China | - | - | - | - | | - | | - | | | - |
| 116 | HM448834.1/fox/2009/China | - | - | - | - | | - | | - | | | - |
| 117 | EU934233.1/raccoondog/2006/China | - | - | - | - | | - | | - | | | - |
| 118 | EU325726.1/raccoondog/2006/China | - | - | - | - | | - | | - | | | - |
| 119 | EF445051.1/fox/2007/China | - | - | - | - | | - | | - | | | - |
| 120 | EF042818.1/raccoondog/2006/China | - | - | - | - | | - | | - | | | - |
| 121 | EU325729.1/raccoondog/2007/China | - | - | - | - | | - | | - | | | - |
| 122 | HM448833.1/raccoondog/2009/China | - | - | - | - | | - | | - | | | - |
| 123 | HM448830.1/raccoondog/2009/China | - | - | - | - | | - | | - | | | - |
| 124 | EU325730.1/raccoondog/2007/China | - | - | - | - | | - | | - | | | - |
| 125 | FJ810215.1/fox/2008/China | - | - | - | - | | - | | - | | | - |
| 126 | EF445054.1/raccoondog/2007/China | - | - | - | - | | - | | - | | | - |
| 127 | EU325727.1/raccoondog/2007/China | - | - | - | - | | - | | - | | | - |
| 128 | HQ128601.1/raccoondog/2010/China | - | - | - | - | | - | | - | | | - |
| 129 | FJ810214.1/raccoondog/2008/China | - | - | - | - | | - | | - | | | - |
| 130 | AB605890.1/raccoondog/2008/Japan | G | - | H | - | | - | | - | | | - |
| 131 | AB619775.1/raccoondog/2009/Japan | - | - | - | - | | - | | - | | | - |
| 132 | AB605891.1/raccoondog/2007/Japan | G | - | H | - | | - | | - | | | - |
|  | **Non-canid** |  |  |  |  | |  | |  | | |  |
| 133 | EU325724.1/mink/2007/China | R | G | Y | V | | L | | Y | | | Y |
| 134 | EU325723.1/mink/2007/China | - | - | - | - | | - | | - | | | - |
| 135 | EU379560.1/mink/2007/China | - | - | H | - | | - | | - | | | - |
| 136 | EU325731.1/mink/2007/China | - | - | - | - | | - | | - | | | - |
| 137 | EU325725.1/mink/2006/China | - | - | - | - | | - | | - | | | - |
| 138 | AB619774.1/tiger/2010/Japan | - | - | - | - | | - | | - | | | - |
|  | **ASIA II** |  |  |  |  | |  | |  | | |  |
|  | **Domestic dog** |  |  |  |  | |  | |  | | |  |
| 139 | EU716073.1/dog/1997/SouthKorea | R | E | Y | V | | L | | Y | | | Y |
| 140 | EU716075.1/dog/2007/SouthKorea | - | G | - | - | | - | | - | | | - |
| 141 | AB025270.1/dog/1999/Japan | G | - | - | - | | - | | - | | | - |
| 142 | AB040767/dog/2000/Japan | - | - | - | - | | - | | - | | | - |
|  | **Non-canid** |  |  |  |  | |  | |  | | |  |
| 143 | EU716074.1/marten/1998/SouthKorea | R | G | Y | V | | L | | Y | | | Y |
|  | **EUROPE** |  |  |  |  | |  | |  | | |  |
|  | **Domestic dog** |  |  |  |  | |  | |  | | |  |
| 144 | HM563059.1/dog/2007/Portugal | R | G | Y | V | | L | | Y | | | Y |
| 145 | GQ214376.2/dog/2002/Austria | - | - | - | - | | - | | - | | | - |
| 146 | GQ214378.2/dog/2002/Austria | - | - | - | - | | - | | - | | | - |
| 147 | DQ494317.1/dog/2006/Italy | - | - | - | - | | - | | - | | | - |
| 148 | DQ494319.1/dog/2006/Italy | - | - | - | - | | - | | - | | | - |
| 149 | DQ494318.1/dog/2006/Italy | - | - | - | - | | - | | - | | | - |
| 150 | DQ889177.1/dog/2006/Hungary | - | - | - | - | | - | | - | | | - |
|  | **Wild canid** |  |  |  |  | |  | |  | | |  |
| 151 | HM563057.1/wolf/1998/Portugal | R | G | Y | V | | L | | Y | | | Y |
| 152 | HM563058.1/wolf/2008/Portugal | - | - | - | - | | - | | - | | | - |
| 153 | FJ416339.1/fox/2008/Germany | - | - | H | - | | - | | - | | | - |
| 154 | JN153025.1/redfox/2008/Germany | - | - | - | - | | - | | - | | | - |
| 155 | JN153024.1/redfox/2008/Germany | - | - | - | - | | - | | - | | | - |
| 156 | HM120874.1/redfox/2009/Italy | - | - | H | - | | - | | - | | | - |
|  | **Non-canid** |  |  |  |  | |  | |  | | |  |
| 157 | FJ416338.1/badger/2008/Germany | R | G | H | V | | L | | Y | | | Y |
| 158 | GU001863.1/Iberianlynx/2005/Spain | - | - | - | - | | - | | - | | | - |
| 159 | GU001864.1/Iberianlynx/2005/Spain | - | - | - | - | | - | | - | | | - |
|  | **EUROPE WILDLIFE** |  |  |  |  | |  | |  | | |  |
|  | **Domestic dog** |  |  |  |  | |  | |  | | |  |
| 160 | DQ228166.1/dog/2005/Italy | R | N | H | V | | L | | Y | | | Y |
|  | **Non-canid** |  |  |  |  | |  | |  | | |  |
| 161 | JN153020.1/raccoon/2007/Germany | R | D | H | V | | L | | Y | | | Y |
| 162 | JN153023.1/raccoon/2007/Germany | - | - | - | - | | - | | - | | | - |
| 163 | GQ214374.2/badger/2006/Austria | - | - | - | - | | - | | - | | | - |
| 164 | GQ214369.2/stonemartin/2007/Austria | - | - | - | - | | - | | - | | | - |
|  | **SOUTH AMERICA I / EUROPE** |  |  |  |  | |  | |  | | |  |
|  | **Domestic dog** |  |  |  |  | |  | |  | | |  |
| 165 | FJ392652.1/dog/2003/Argentina | R | G | Y | V | | L | | Y | | | Y |
| 166 | JN215476.1/dog/2009/Uruguay | - | - | - | - | | - | | - | | | - |
| 167 | JN215475.1/dog/2008/Uruguay | - | - | - | - | | - | | - | | | - |
| 168 | JN215473.1/dog/2007/Uruguay | - | - | - | - | | - | | - | | | - |
| 169 | JN215477.1/dog/2009/Uruguay | - | - | - | - | | - | | - | | | - |
| 170 | JN215474.1/dog/2008/Uruguay | - | - | - | - | | - | | - | | | - |
| 171 | EU098105.1/dog/2007/Brazil | - | S | - | - | | - | | - | | | - |
| 172 | EU098103.1/dog/2007/Brazil | - | S | - | - | | - | | - | | | - |
| 173 | EU098104.1/dog/2007/Brazil | - | S | - | - | | - | | - | | | - |
| 174 | EU098102.1/dog/2007/Brazil | - | - | - | - | | - | | - | | | - |
|  | **SOUTH AMERICA II** |  |  |  |  | |  | |  | | |  |
|  | **Domestic dog** |  |  |  |  | |  | |  | | |  |
| 175 | FJ392651.1/dog/2005/Argentina | R | D | Y | V | | L | | Y | | | Y |
| 176 | KC257464.1/dog/2010/Argentina | - | - | - | - | | - | | - | | | - |
| 178 | FJ011005.1/dog/2005/Argentina | - | - | - | - | | - | | - | | | - |
|  | **SOUTH AMERICA III** |  |  |  |  | |  | |  | | |  |
|  | **Domestic dog** |  |  |  |  | |  | |  | | |  |
| 179 | KF835411/dog/2012/Columbia | R | N | Y | V | | L | | Y | | | Y |
| 180 | KF835412/dog/2012/Columbia | - | - | - | - | | - | | - | | | - |
| 181 | KF835413/dog/2012/Columbia | - | - | - | - | | - | | - | | | - |
| 182 | KF835414/dog/2012/Columbia | - | - | - | - | | - | | - | | | - |
| 183 | KF835420/dog/2012/Columbia | - | - | - | - | | - | | - | | | - |
| 184 | KF835423/dog/2012/Columbia | - | - | - | - | | - | | - | | | - |
| 185 | KF835425/dog/2012/Columbia | - | - | - | - | | - | | - | | | - |
|  | **NORTH AMERICA** |  |  |  |  | |  | |  | | |  |
|  | **Domestic dog** |  |  |  |  | |  | |  | | |  |
| 186 | KJ747372/dog/2010/USA | R | D | Y | V | | L | | Y | | | Y |
|  | **Wild canid** |  |  |  |  | |  | |  | | |  |
| 187 | KJ747371/fox/2010/USA | R | D | Y | V | | L | | Y | | | Y |
